# Progress in Reaction Mechanisms and Reactor Technologies for Thermochemical Recycling of Poly(methyl methacrylate)

**DOI:** 10.3390/polym12081667

**Published:** 2020-07-27

**Authors:** Eli K.C. Moens, Kyann De Smit, Yoshi W. Marien, Alessandro D. Trigilio, Paul H.M. Van Steenberge, Kevin M. Van Geem, Jean-Luc Dubois, Dagmar R. D’hooge

**Affiliations:** 1 Laboratory for Chemical Technology, Ghent University, Technologiepark 125, 9052 Ghent, Belgium; Eli.Moens@UGent.be (E.K.C.M.); Kyann.DeSmit@UGent.be (K.D.S.); Yoshi.Marien@UGent.be (Y.W.M.); Alessandro.Trigilio@UGent.be (A.D.T.); Kevin.VanGeem@UGent.be (K.M.V.G.); Dagmar.Dhooge@UGent.be (D.R.D.); 2 Arkema France, 420 Rue d’Estienne d’Orves, F-92705 Colombes, France; jean-luc.dubois@arkema.com; 3 Department of Materials, Textiles and Chemical Engineering, Ghent University, Technologiepark 7Oa, 9052 Ghent, Belgium

**Keywords:** polyacrylics, thermochemical recycling, degradation mechanisms, reactor technologies, polymer circularity

## Abstract

Chemical or feedstock recycling of poly(methyl methacrylate) (PMMA) by thermal degradation is an important societal challenge to enable polymer circularity. The annual PMMA world production capacity is over 2.4 × 10^6^ tons, but currently only 3.0 × 10^4^ tons are collected and recycled in Europe each year. Despite the rather simple chemical structure of MMA, a debate still exists on the possible PMMA degradation mechanisms and only basic batch and continuous reactor technologies have been developed, without significant knowledge of the decomposition chemistry or the multiphase nature of the reaction mixture. It is demonstrated in this review that it is essential to link PMMA thermochemical recycling with the PMMA synthesis as certain structural defects from the synthesis step are affecting the nature and relevance of the subsequent degradation reaction mechanisms. Here, random fission plays a key role, specifically for PMMA made by anionic polymerization. It is further highlighted that kinetic modeling tools are useful to further unravel the dominant PMMA degradation mechanisms. A novel distinction is made between global conversion or average chain length models, on the one hand, and elementary reaction step-based models on the other hand. It is put forward that only by the dedicated development of the latter models, the temporal evolution of degradation product spectra under specific chemical recycling conditions will become possible, making reactor design no longer an art but a science.

## 1. Introduction

Today recycled polymers account for only 6% of the total polymer amount in Europe [1,2]. Low prices and uncertainties about market outlets have initially led to prospects of low profitability and therefore depressed investments in new polymer recycling capacity in the last decades, causing the EU polymer recycling sector to suffer [2]. Yet with the present common societal sense regarding to need of sustainable chemistries with a minimization of the environmental footprint, corporate interest and government funding is increasing regarding research projects focusing on recycling of polymers and making the polymer industry more sustainable [1]. Recycling of polymer waste is the most desired choice with respect to sustainability, compared to simply relying on depleting fossil fuel resources.

**Figure 1:**
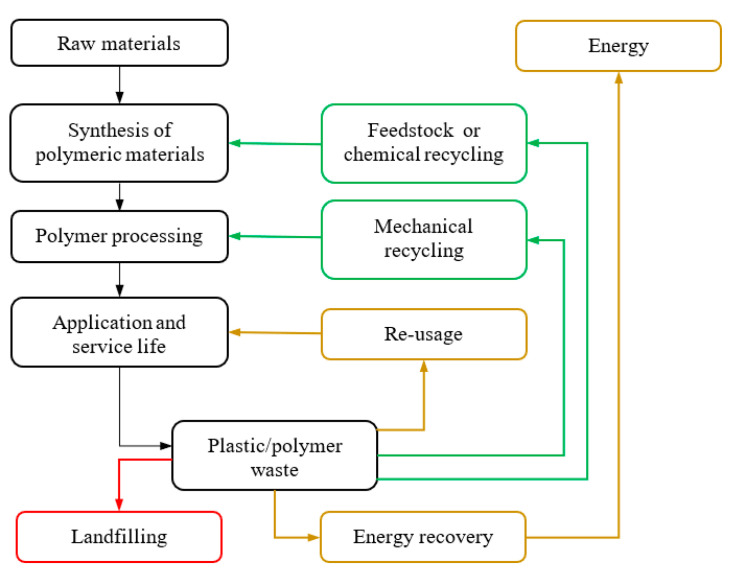
Representation of plastic/polymer waste management based on the work of Vilaplana and Karlsson [3]. Colors are guide of the eye regarding the desired implementation; red: undesired; brown: to be minimized; green: preferred.

In Figure 1, the main types of polymer waste treatment are shown [3]. Landfilling should be avoided, and energy recovery and direct re-use minimized so that focus can be on mechanical recycling on the one hand and feedstock or so-called chemical recycling on the other hand. Mechanical recycling is the process of recovering solid plastic waste (SPW) for repeated polymer manufacturing via mechanical means, such as (non-reactive) extrusion technology. Already in the 1970s, mechanical recycling, which is also known as secondary recycling, was promoted and commercialized. It is therefore one of the most common methods of SPW recycling and relatively well-known. Mechanical recycling is, however, only applicable for well-defined basic waste streams ideally composed of one (linear or loosely crosslinked) polymeric component such as polypropylene (PP), polyethylene (PE) and polystyrene (PS). Furthermore, the degree of SPW contamination has a significant effect on the practicality of mechanical recycling to achieve the desired product quality. Costly unit operations such as separation, washing and preparation are all essential to produce high quality, clear, clean and homogeneous end-products, explaining why, next to mechanical recycling, often incineration for energy recovery is still considered [4,5]. 

Chemical or feedstock recycling, which is also known as tertiary recycling, refers to chemical modification routes which convert polymeric materials back into smaller molecules, in the limit the original monomer(s). The products are usually a mixture of liquids and gasses which can serve as a feedstock for the production of new chemicals, fuels and polymers [4]. Important chemical processes are pyrolysis, gasification, liquid-gas hydrogenation, viscosity breaking, reactive extrusion and steam or catalytic cracking [6,7,8,9,10]. Significant focus has been put on thermochemical recycling of vinyl polymers, such as polyolefins producing a mixture of numerous components, which can be used as fuels. Condensation polymers, such as polyethylene terephthalate and nylon, can also be subjected to chemical recycling to produce oligomers that can be subsequently converted in high-added value building blocks [11]. SPW is also used as a reducing agent in blast furnaces to enable sustainable production of iron [12]. More recently, polymers are also chemically modified in their original synthesis route to enable controlled degradation or re-shaping at the end-of-life [13,14,15,16,17,18].

In this review, the interest goes to the thermal thus non-catalytic chemical recycling of poly(methyl methacrylate) (PMMA), an important vinyl polymer. Mechanical recycling of PMMA is less desired as the resulting product often does not lead to the desired optical properties. Kikuchi et al. reported that chemical recycling of PMMA can further reduce the environmental impact when mechanical recycling has limited potential [19]. In contrast to most other polymers, it is possible to quantitatively depolymerize pure PMMA towards the original monomer [20]. Already at the start of the large-scale industrial production of PMMA, cracking of PMMA towards MMA has been investigated. In these earlier processes, PMMA was mixed with sand or with super-heated steam to prevent uncontrolled pyrolysis and was performed in a batch wise process configuration. More recently, more economically feasible continuous processes were developed, in which use is made of an indirectly heated fluidized bed reactor environment [21,22,23].

In 2017, a global capacity of 2.4 × 10^6^ tons was reached, but currently in Europe only 3.0 × 10^4^ tons of PMMA is collected and recycled, highlighting the opportunities in the field of recycling of PMMA and taking into account that the process energy needed for virgin MMA synthesis is relatively high [24,25,26]. By developing new engineering routes, making use of chemically recycled MMA and advanced process design, the process energy demand might be reduced. In the scientific literature, relatively limited focus has however been put on the studying of PMMA chemical recycling reactor technology with low residence times and high MMA yields. It should be further realized that depolymerization chemistry is also less studied than polymerization chemistry, the latter conducted at much lower temperatures (e.g., 353 compared to 553 K) with, as rule as thumb, fewer side reactions. Even for a simple structure, such as a MMA unit in a polymer chain, a debate still exists in the literature regarding the dominance of specific reaction pathways in chemical recycling, as several structural defect types can be created during synthesis or further modification [27,28,29,30,31,32,33]. 

PMMA synthesis was already developed from 1928 onwards by several research groups and brought to the market in 1933 by Germans Röhm and Haas AG, under the trademark of Plexiglas [20,34]. PMMA can be seen as an economical alternative to polycarbonate (PC) as soon as impact strength becomes less important than the optical and mechanical properties. An advantage of PMMA, compared to PC, is that it does not contain bisphenol A, which is a hazardous toxic chemical [35]. The most used process by European producers of the monomer MMA is the acetone cyanohydrin route [24,36]. This process can be subdivided in three consecutive steps. The first step is the production of hydrogen cyanide out of ammonia and methane. This is realized via the Andrussow process or the Degussa process [37,38]. In a second step, acetone is brought in contact with the hydrogen cyanide with the aim of producing acetone cyanohydrin. In a third step the formed acetone cyanohydrin reacts with an excess of sulfuric acid which leads to the formation of methacrylamide sulfate. The methacrylamide sulfate is subsequently brought into contact with an excess of aqueous methanol, resulting in the formation of MMA and ammonium sulfate. Normally, sulfuric acid can be recycled or neutralized with ammonia, producing ammonium sulfate as co-product which can be, together with the formed ammonium sulfate from the MMA production, sold to the fertilizer market [36].

**Figure 2:**
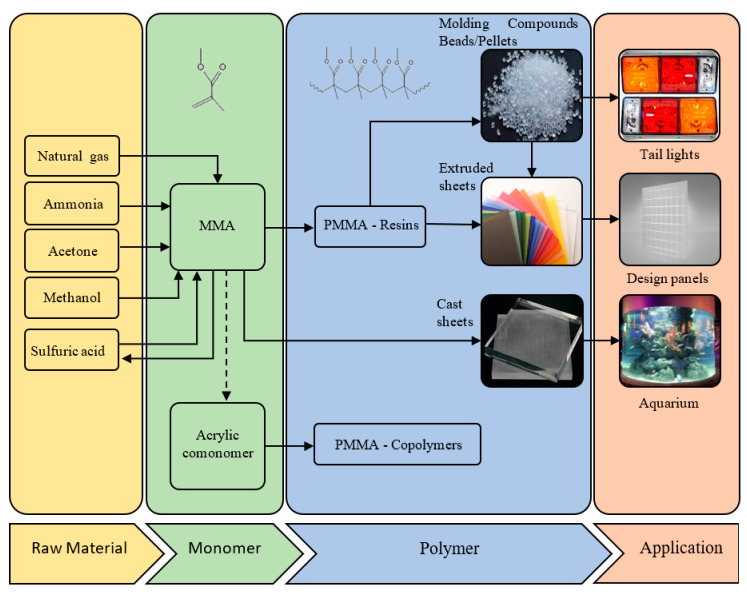
Overview of the synthesis of poly(methyl acrylate) (PMMA); starting from raw material and its applications based on the work of the Methacrylate Sector Group [40].

Nowadays, PMMA can be found in many sectors ranging from the automotive, construction and healthcare industries to the electronics industry, as illustrated in Figure 2. Here, a distinction can be made between PMMA sheets obtained via direct casting or via extrusion. To meet the different material property requirements of the PMMA product, depending on its application, more recent developments made clear that additives need to be included in the formulation or the PMMA backbone needs to be modified. For example, small amounts of acrylate comonomer have been incorporated to increase the thermal resistance of MMA-rich polymers during extrusion and to control the flexibility of the material [39]. Hence, a transition from homopolymers to MMA rich copolymers has been established, complicating the chemical recycling as more reaction possibilities are expected with possibly different optimal reactor settings depending on the (co)polymer reactant type. 

In what follows, an overview of the state-of-the-art depolymerization/degradation reaction schemes and reactor technologies for PMMA thermal chemical recycling is given. A first distinction is made between PMMA synthesized via anionic polymerization (AP) and conventional free radical polymerization (FRP), explaining why the review commences with PMMA synthesis molecular parameters and reaction mechanisms. A second distinction is made between depolymerization of PMMA as such and copolymers with as major monomer unit MMA. The focus is both on experimental analysis and (kinetic) modeling tools, to understand the interplay of chemical reactions.

## 2. Radical and Anionic Polymerization Reaction Mechanisms for PMMA Synthesis 

The synthesis of PMMA is realized in many cases by FRP of MMA. Depending on the application and the desired properties, different reaction media can be considered. In this review, the main focus is on feedstock recycling of PMMA products originating from FRP of MMA in bulk. FRP of MMA follows a typical chain-growth mechanism involving in essence four consecutive types of chemical reactions, i.e., dissociation, chain initiation, propagation and termination, augmented by side reactions, such as chain transfer to monomer [41,42,43]. The propagation rate is many orders of magnitude larger than the dissociation, chain initiation and termination rate, the latter rates being similarly low thereby leading to a quasi-steady state for the concentration of the macroradicals. PMMA chains with a typical number average chain length above 10^3^ are formed on the timescale of ca. 1 s or less at a typical polymerization temperature of 333 to 393 K [44]. As radical synthesis is highly random, high dispersity values are possible, with typical values between 1.5 and 2 but also higher [43].

Alternatively, PMMA can be obtained by AP, in which anions are the chain carriers, as opposed to radicals in FRP. Here, the number average chain length is smaller with typical values below 10^2^, the polymerization temperature is only for instance 195 K and solution polymerization conditions are considered [45]. Furthermore, the dispersity is lower (in the limit a value of 1), inherent to the faster initiation of AP on a chain basis, compared to FRP and the absence of termination reactions, despite that much more stringent reaction conditions are required.

In what follows, first, the free radical polymerization mechanism is discussed in detail, with specific focus on the aspects relevant for recycling/degradation, such as the formation of specific structural defects from which degradation can be started more easily. A similar discussion is subsequently provided for the AP mechanism.

### 2.1. Radical Polymerization Mechanism 

**Figure 3:**
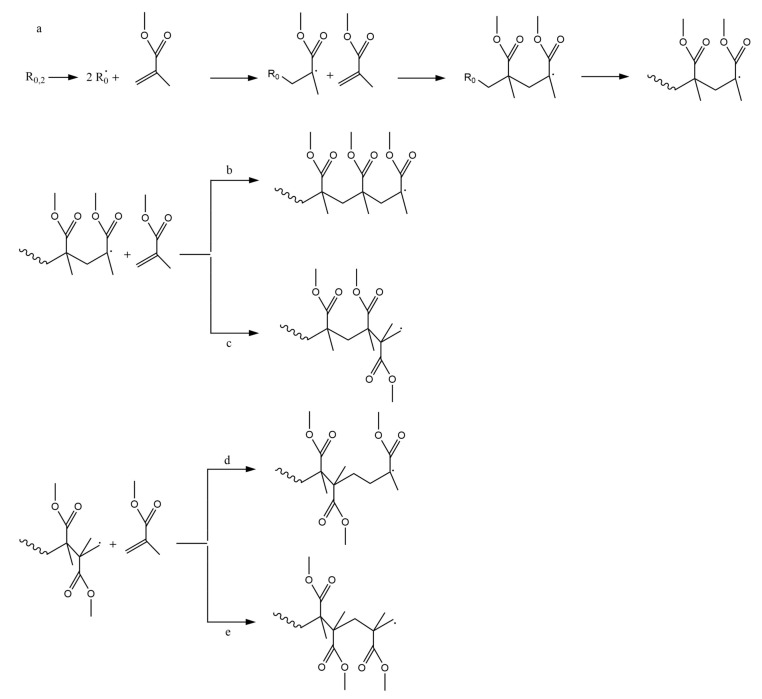
(**a**) Initiator dissociation, chain initiation and propagation as basic reactions in free radical polymerization (FRP) of methyl methacrylate (MMA); (**b**) four types of propagation (addition) for FRP of MMA: head-to-tail (H–T), head-to-head (H–H) (**c**), tail-to-tail (T–T) (**d**) and tail-to-head (T–H) (**e**) addition. H–T is the most common addition so that H–H and T–T linkage or dyads must be seen as structural defects.

Initiator dissociation is a reaction in which an initiator species R_0,2_ converts into one or more initiator radicals R_0_. Depending on the chemical nature and stability of the initiator used, the fragmentation of the initiator can be realized under influence of heat, light or even just over time [46,47,48,49]. The initiator radicals can add to the double bond of a first MMA (in general monomer (M)) species present, forming an oligoradical of chain length one, defining the so-called chain initiation step. Upon addition of several monomers, oligoradicals convert into macroradicals, as illustrated in Figure 3a. Note that some initiator radicals formed via initiator dissociation might not lead to chain initiation. Hence, in general, initiator efficiency needs to be considered, defining the fraction of initiator radicals actually leading to chain initiation [50,51]. At elevated temperature, self-initiation can also take place [52].

For 1,1-disubstituted vinyl monomers, such as MMA, it is commonly accepted to call the less substituted part (CH_2_) the “tail” and the more substituted part (CX_1_X_2_; X_1_=CH_3_ and X_2_=COOCH_3_ for MMA), the “head” of the monomer [43,53]. This allows to define four propagation (or addition) reactions as illustrated in Figure 3b–e: head-to-tail (H–T), head-to-head (H–H), tail-to-tail (T–T) and tail-to-head (T–H), leading, respectively, to H–T, H–H, T–T and T–H linkages or dyads. Head radicals in FRP of MMA show the tendency to react via H–T addition, as then a tertiary instead of primary macroradical is formed, so that H–H and T–T linkages must be seen as structural defects. In other words, if H–H propagation occurs one can expect the most probable next propagation to be a T–T addition as a primary radical wants to swap back to a more stable tertiary radical [43]. 

**Figure 4:**
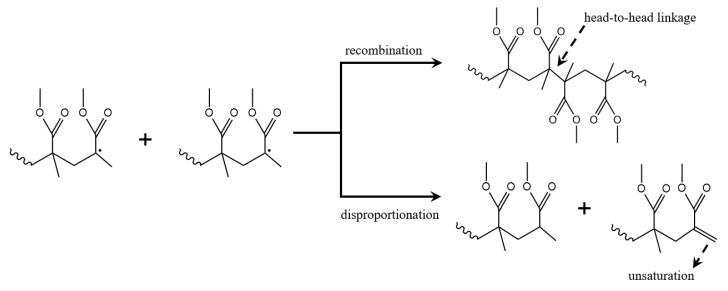
Top: termination by recombination of two head PMMA macroradicals, giving rise to the formation of head-to-head linkages or structural defects; Bottom: termination by disproportionation of two head PMMA macroradicals leading to the formation of unsaturations.

The last step in the radical mechanism is the formation of “dead” polymer chains, the removal of radical centers by a chain growth stopping event. In this respect, the PMMA macroradicals may react with another growing macroradical or with an added terminator or radical scavenger. Most commonly focus is on termination between two macroradicals, as illustrated in Figure 4 considering two head radicals. If termination of such two radicals occurs via (re)combination, a H–H linkage is formed. If termination via disproportionation or equivalently β-H abstraction occurs, two structurally different dead polymer chains are formed, i.e., one with an unsaturated and one with a saturated chain end. The disproportionation contribution has been reported to be above 80% [28,54], although detailed numbers are still a point of discussion.

Figure 4 gives example of chain growth stopping events where a distinction is made between termination by recombination or by disproportionation. These reactions complete a typical cycle in FRP starting with the reactions in Figure 3. Several studies have indicated that the observed termination reactivity is chain length dependent and decreasing with increasing monomer conversion [55,56,57,58,59,60,61]. This decrease is caused by diffusional limitations as macroradicals have a restricted mobility specifically if they become longer or embedded in a more viscous polymer matrix. Hence, the kinetics of FRP need to be described with apparent termination rate coefficients, instead of intrinsic ones, i.e., a gel-effect needs to be accounted for as evidenced by an increase in polymerization rate and average chain length compared to the situation of (theoretical) intrinsic kinetics [62]. Chain length dependencies also exist for other reactions, but are typically intrinsic and often limited to the lower chain lengths, as is the case for propagation and chain transfer reactions [63,64]. At higher monomer conversions one should account for a potential cage and glass effect which, respectively, correspond to diffusional limitations on radical initiator dissociation and propagation [65].

Inspection of Figure 3c and Figure 4 together highlights the chemical distinction between H–H linkages formed during propagation and those formed by termination by recombination because their adjacent dyads are different. H–H linkages originating from propagation are contained in H–(H–H)–T triads, whereas H–H linkages originating from recombination are contained in T–(H–H)–T triads. It should be realized that, with spectroscopic analysis, it is difficult to distinguish between the various types of H–H linkages, as the overall chemical environments are similar and overlapping signals for high chain length species are inevitable. In contrast, it can be expected that a clearer distinction can be made based on whether or not T–T linkages are present. However, so far, limited effort has been paid to applying spectroscopic methods detecting T–T linkages [43]. 

**Figure 5:**
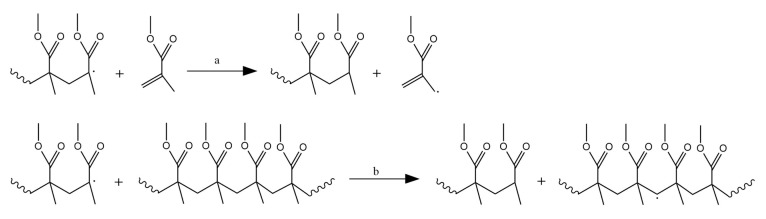
Additional reactions in free radical polymerization (FRP) of methyl methacrylate as opposed to the basic description displayed in Figure 3; focus on (**a**) chain transfer to monomer and (**b**) chain transfer to polymer twice with a head radical. The chain transfer to polymer reaction is however typically ignored.

Figure 5 shows examples of additional reactions in PMMA synthesis via a radical mechanism. The focus is on chain transfer to monomer creating a dead chain, but also maintaining the chain growth as a small monomeric radical is formed. Chain transfer to dead polymer or intermolecular hydrogen abstraction is also shown, in which mid-chain radicals are formed and further propagation leads to branch formation. Intermolecular hydrogen abstractions are mostly relevant at higher monomer conversions and if large chain lengths are present, but these are typically not reported to occur for radical polymerization of MMA [10].

It should be also noted that in case a second comonomer type (e.g., n-butyl acrylate (nBuA) next to MMA) is present the number of reaction possibilities increases as one can form several monomer sequences and types of termination products. In practice, it is advisable to identify the most dominant reaction steps, taking into account that more recent research has indicated the relevance of penultimate monomer unit (PMU) effects as well [66]. This implies that, to describe the reactivity of a macroradical, one should potentially also consider the monomer unit preceding the unit containing the radical center (the so-called penultimate monomer unit), instead of a conventional approach focusing only on the terminal monomer unit: hence, the unit with the radical center [67,68,69].

### 2.2. Anionic Polymerization Mechanism 

**Figure 6:**
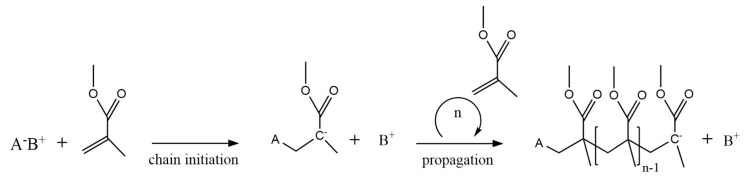
Reaction mechanism for anionic polymerization (AP) of MMA.

The reaction scheme for AP of MMA is much more straightforward, as illustrated in Figure 6. AP is suitable for the polymerization of vinyl monomers with strongly electronegative substituents. AP initiators (A^−^B^+^ species in Figure 6) are either electron transfer agents or strong anions which transfer an electron to an MMA molecule. This gives rise to chain initiation forming an oligo-anion of chain length 1 and ultimately macro-anions. Propagation in AP occurs (almost) exclusively via H–T propagation due to the attractive force of opposite (partial) charges. In the absence of impurities, such as traces of water, AP is considered to follow a living polymerization mechanism, meaning that the macro-anions remain indefinitely active unless there is unintended or deliberate termination or chain transfer. Hence, no H–H linkages are in principle formed during AP of MMA.

## 3. Radical Reaction Mechanisms for Thermal PMMA Chemical Recycling 

**Figure 7:**
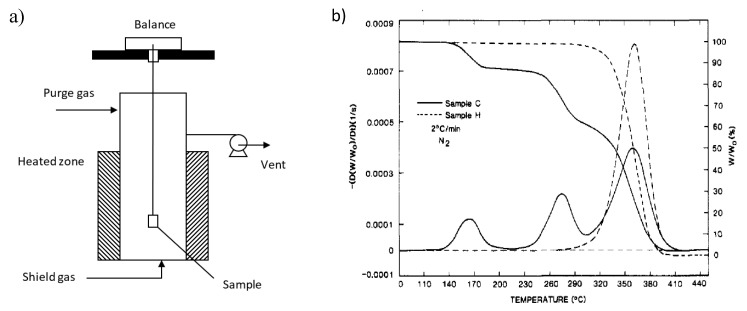
(**a**) Schematic representation of thermogravimetric analysis (TGA) instrument, (**b**) more complex overall degradation mechanism for poly(methyl methacrylate) (PMMA) made by free radical polymerization (FRP) as compared to PMMA made by anionic polymerization (AP); comparison of mass loss (here symbol W) for sample C (FRP) and sample H (AP) using TGA and differential thermal analysis (DTG) analysis; reproduced with the permission from ACS [28].

Kashiwagi et al. proposed a three-stage thermal degradation mechanism of PMMA synthesized via FRP and a one stage thermal degradation mechanism for PMMA obtained via AP [28]. This proposition is based on thermogravimetric analysis (TGA), which allows to measure the mass loss of PMMA lab-scale samples, with respect to a predefined temperature program, and to identify peaks by taking the derivative, with respect to temperature through so-called differential thermal gravimetric (DTG) analysis [70]. More in detail, in TGA a small sample is placed in an oven, as depicted in Figure 7a. There, the sample is submitted to a temperature profile at which decomposition takes place. During the degradation, volatile species are formed which are entrained from the oven by a circulating inert gas. The volatilization of these gaseous species gives rise to mass loss which is measured by the balance on which the sample is placed inside the oven.

As shown in Figure 7b, for FRP made MMA, three peaks are obtained in contrast to the AP degradation case displaying one peak. This difference in the number of peaks (three vs. one) highlights the more complex degradation of PMMA made via FRP, consistent with the higher number of reactions and structural defects as discussed in the previous section. Note that also spectra with more than three peaks have been recorded for FRP-made MMA, further confirming the above statements.

In what follows, the degradation mechanisms are defined from an elementary reaction point of view and they are unless stated otherwise all of a radical nature. A main distinction is first made between reactions leading to initiation, depropagation and termination, so that their sequence leads to a closed cycle and thus a degradation mechanism. Initiation reactions are at first defined by the occurrence of a fission, in which a polymer chain without active centers is submitted to a homolytic bond cleavage, resulting in the formation of two smaller macroradicals that are in most cases end-chain radicals. Alternatively, an impurity or trace amounts of molecular oxygen can induce the formation of mid-chain radicals for instance by hydrogen abstraction. Further reaction of these mid-chain radicals can then start an actual degradation. Depropagation relates to a β-scission of species with an active center and forms unsaturated smaller species. Termination reactions are similar as in the PMMA synthesis, although a spectrum of smaller chain lengths is eventually expected. As thermal degradation is conducted at elevated temperature, many side reactions blocking the ultimate formation of MMA can be identified as well. These types of reactions are discussed at the end of this section as well.

### 3.1. Initiation by Head-Tail Fission 

**Figure 8:**
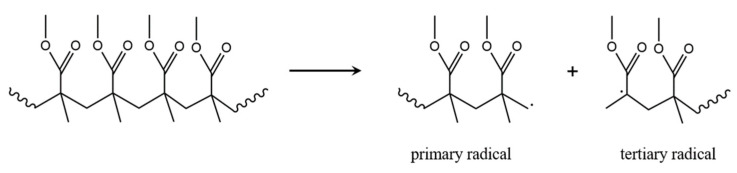
First thermal degradation initiation reaction by random fission of poly(methyl methacrylate) PMMA (so at head-tail structural characteristic formed during synthesis) with the formation of a primary and tertiary macroradical.

The main thermal initiation degradation reaction at high temperature is generally accepted to be random fission and thus defined based on the dominantly present H–T linkages as formed during PMMA synthesis (see Section 2) [29,71]. During such H–T fission, as shown in Figure 8, a primary and a tertiary macroradical are formed [72]. Note that AP-made PMMA only consists out of H–T linkages, and thus a single initiation mechanism is expected, consistent with the TGA/DTG profile in Figure 7b. 

Speculation exists whether H–T fission takes place in the early stages of thermal degradation of PMMA. It has been postulated that this initiation reaction is kinetically limited by a so-called cage effect, in which the restricted mobility of the macroradicals in the reaction mixture (e.g., melt) causes them to recombine and reform the initial H–T linkage before actual depropagation takes place [30,31,33]. On the other hand, H–T fission is kinetically favored by the presence of many such linkages in the starting product.

### 3.2. Initiation by Head-Head Fission 

**Figure 9:**
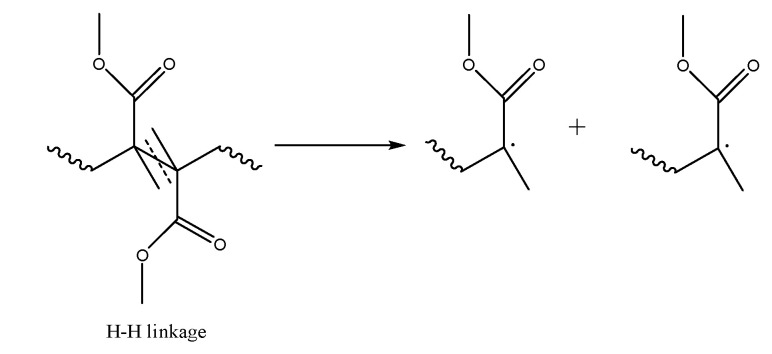
Second thermal degradation initiation reaction via fission at the head-head linkage formed 3.3. Initiation by chain-end fission.

A second thermal degradation initiation reaction involves the weaker H–H linkages, as present in FRP-made PMMA. Fission leads here to the formation of two tertiary macro-radicals, as depicted in Figure 9. It can be expected based on typical rate coefficients that the H–H linkages in FRP made PMMA originate predominantly from termination via recombination reactions and, to a lesser extent, from H–H propagation reactions [43]. In addition, the assumption that H–H linkages are only formed in FRP is consistent with the absence of a peak at low temperatures consistent with breakages of weaker bonds in de DTG profile of PMMA made by AP in Figure 7b. It should be noted that also here a cage effect could be active, although in the original work of Manring most focus was on H–T fission. 

**Figure 10:**
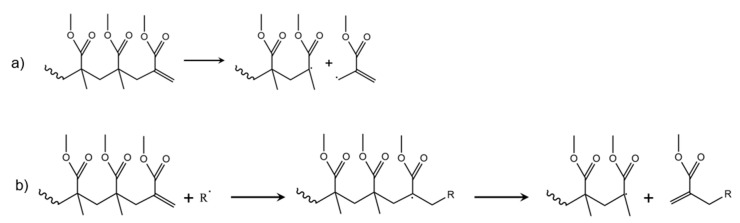
(**a**) Third thermal degradation initiation reaction based on chain-end fission thus involving the unsaturation as formed through termination by disproportionation during the synthesis; (**b**) Alternative based on addition of a general radical R, as introduced by Manring [30]. Here the initial and subsequent reaction (β-scission) are depicted as their combination leads to a degradation initiation.

As shown in Figure 10a, a third thermal degradation initiation reaction can be defined as chain-end fission. In this case, bond cleavage takes place at an unsaturated double bond, possibly located at the PMMA chain-end. This happens only on the condition that disproportionation has taken place during the synthesis, which is not the case for PMMA made by AP, as confirmed in Figure 7b, with only one peak for a higher temperature phenomenon. This type of initiation has been observed in TGA/DTG analysis for FRP-made polymers at elevated temperatures between 573 and 593 K, thus lower than the temperature needed for random or H–T fission (600–675 K) [28,73]. The reason why this reaction occurs at lower temperature is because a tertiary and an allylic radical are formed, with the latter radical more stable than the primary radical formed in H–T fission [30]. 

However, Manring indicated a different role of the vinyl-terminated PMMA [30]. A radical in the reaction mixture adds to the double bond of the unsaturated unit of PMMA, as shown in Figure 10b (first step), leading to the formation of a tertiary macroradical, which is prone to depropagation (second step in the same subplot).

### 3.3. Initiation by Side-Group Fission 

**Figure 11:**
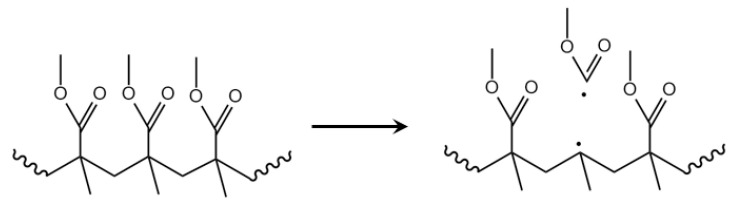
Fourth thermal degradation initiation reaction based on side-group fission with the methoxy carbonyl group [32].

Manring postulated a fourth thermal degradation initiation reaction which involves fission of the methoxy carbonyl side group of PMMA and the formation of a mid-chain radical, as shown in Figure 11 [32]. He put forward, as already explained above, that H–T (and H–H) fission can become kinetically inhibited relative to side-group (or chain-end) fission [31]. He claimed that due to a cage effect too large tertiary radicals cannot diffuse away from each other and instead have almost no other fate than to terminate again so that apparent kinetics are established with even in the limit a zero reaction rate. In contrast, the side-group (or chain-end fission) generates small molecules which are much more mobile, thus enabling effective initiation for degradation. 

### 3.4. Initialization Involving Transfer Reactions or Interactions

Radical transfer reactions can take place leading to the formation of (mid-chain) radicals which, upon further chemical modification, enable initialization of the PMMA degradation, as every hydrogen in PMMA chains can be subject to a hydrogen abstraction event. As this gives rise to a large number of widely different products depending on which hydrogen is abstracted, these reactions are not further elaborated in detail in the scope of this review. It should be noted that impurities could be the reactant of the transfer reaction or these transfer reactions only take place once a certain number of radicals is formed during the degradation.

**Figure 12:**
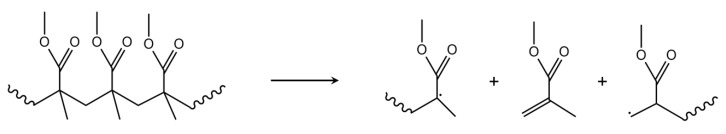
Alternative concerted pathway leading to initiation (so fifth mechanism) in the theoretical research of Stoliarov et al. [74].

Furthermore, Stoliarov et al. implemented reactive molecular dynamics (RMD) to study the degradation mechanism of PMMA in an inert atmosphere [74]. Classical force-field-based molecular dynamics have been adapted to mimic chemical reactions. They observed that neither main chain/backbone or side group fission were the major initiation trigger, with only less than 20% of all initiation events. They claimed an alternative concerted initiation reaction, as depicted in Figure 12. Possible reasons as put forward by these authors are internal and external interactions with different inertia of larger polymer chains and stabilization of the transition state, due to formation of a π bond between the two interior carbons, and covalent interactions between the two end carbons, which eventually become radicals. Yet the remark has to be made that a first order approximation was made for the potential energy surfaces of the chemical reactions. One could additionally interpret that these authors only capture a thermodynamic overall effect of a sequence of reactions ultimately leading to the products in Figure 12.

### 3.5. Depropagation through End-Chain β-Scission

**Figure 13:**
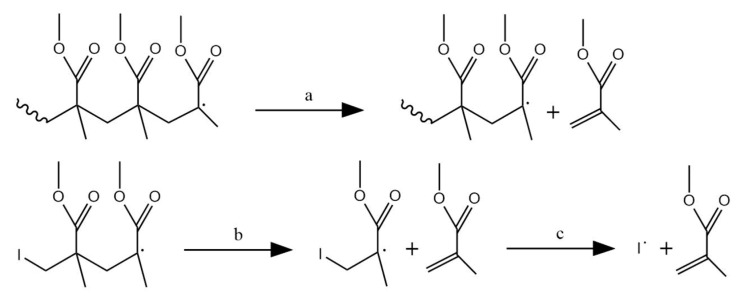
(**a**): Typical end-chain β-scission (“first” type of β-scission) of the tertiary macro-radical formed upon the first or second thermal degradation initiation reaction (see Section 3.1 and Section 3.2); (**b**) repetition of the reaction in (**a**) (so-called unzipping) until the initiator fragment I is retrieved (**c**), so case of complete unzipping (here only shows the last two β-scissions).

Because, under well-defined conditions, the dominating degradation product is MMA, it is commonly accepted that the most frequently occurring degradation reaction rapidly gives rise to the formation of MMA monomer, whereas initiation and termination reactions are markedly slower. Considering the first and second thermal degradation initiation reaction, as introduced above, the resulting tertiary radical can undergo β-chain end scission, as shown in Figure 13a. In case this step is repeated a fast unzipping can take place until the original initiator fragment I is retrieved, as shown in Figure 13b,c.

### 3.6. Depropagation through Mid-Chain β-Scission

**Figure 14:**
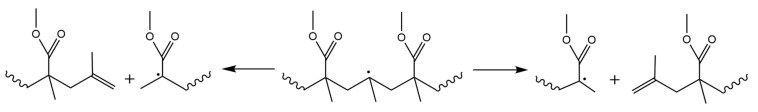
β-scission of the mid-chain radical formed during side-group fission (“second” type of β-scission); shown is a β-scission to the “left” and the “right”.

The tertiary macroradical formed during the side-group fission, as covered in Section 3.5, can also undergo β-scission. To highlight the differentiation with the β-scission in the previous section the term mid-chain β-scission is utilized. As shown in Figure 14, this gives rise to the formation of new radical types and new types of unsaturated macrospecies. Note that these species can undergo similar reactions as already highlighted above (e.g., end-chain β-scission or addition) complicating the overall kinetic description. 

### 3.7. Depropagation through Side Group β-Scission

**Figure 15:**
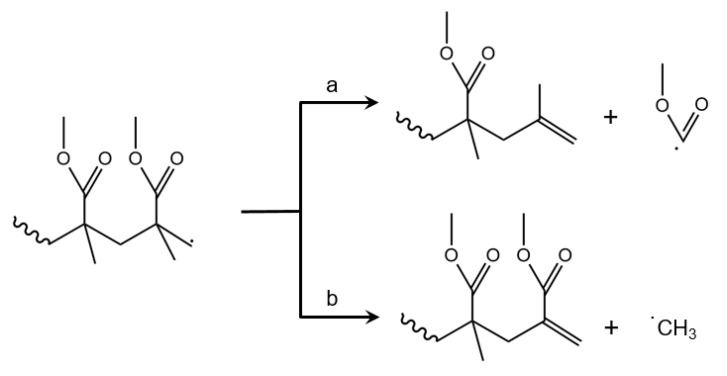
(**a**) Side-group β-scission of the primary radical formed during H–T fission as covered in Section 3.1 (“third” type of β-scission); (**b**) alternative leading to methyl radical formation as proposed in the theoretical research of Stoliarov et al. [74].

Several reports suggest that the primary radical, as formed during H–T fission, further decomposes via a side-group β-scission, as displayed in Figure 15a. The latter reaction leads the formation of an unsaturated PMMA species, which is thus prone to further degradation, but also addition. For completeness, it is mentioned here that Stoliarov et al. theoretically put forward that these primary radicals can also undergo the concerted reaction in Figure 15b [74].

### 3.8. Termination Reactions 

As PMMA depolymerization involves radicals, these radicals may terminate via recombination and disproportionation, as is also the case for the synthesis (cf. Figure 3). It should be reminded that such termination reaction ends a degradation cycle, but still the formed dead polymer molecules can undergo further degradation, provided that they are transformed back into (mid-chain) radicals.

### 3.9. Side Reactions Blocking MMA Formation

**Figure 16:**
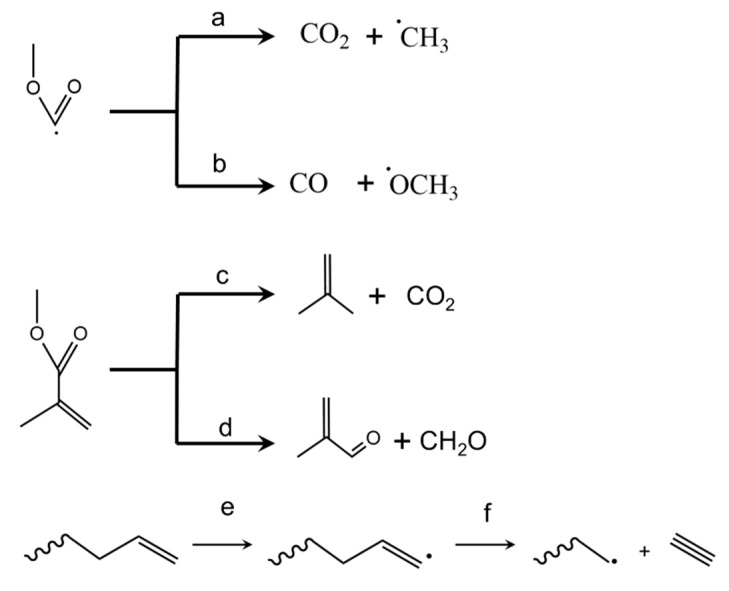
Examples of side reactions blocking MMA formation (**a**,**b**) leading to the formation of neutral CO_2_ and CO as well as OCH_3_ and CH_3_ radicals; note that upon subsequent hydrogen abstraction methanol and methane are formed. (**c**,**d**) direct decomposition products of methyl methacrylate (MMA); (**e**,**f**) formation of acetylene assuming already the removal of CH_3_ groups.

It is interesting to note that methanol and methane have been experimentally observed in the product spectrum from the thermal degradation of PMMA [75,76,77]. Hence, reactions also occur that block eventual MMA formation and must be seen as side reactions. It has been put forward that, after side group β-scission, neutral CO_2_ and CO, as well as OCH_3_ and CH_3_ radicals, are formed, as illustrated in Figure 16a,b. These radicals might abstract a hydrogen from another species to form, respectively, methanol and methane. Furthermore, Burg and Tipper stated that the formation of acetylene (and ethylene) is possible due to specific hydrogen abstraction and follow up β-scission, as shown in Figure 16e,f. In addition, the formation of char has been indicated [73,78]. 

Furthermore, MMA decomposition can take place at elevated temperature, giving rise to the formation of smaller/lighter pyrolysis products. Forman et al. described the thermal degradation of MMA via two scission paths, as shown in Figure 16c,d [79]. The dominant pathway is proposed to be the one with the formation of CO_2_. It is important to mention that the formation of these small products depends heavily on the operation mode for the thermal degradation. If the gaseous MMA is directly removed from the reaction mixture, the timeframe for monomer degradation is relatively small. On the other hand, if the thermal degradation unit is operated in batch mode, the importance of this side reaction might be substantial. 

## 4. From Degradation Reaction Mechanisms for PMMA to MMA-Rich Copolymers with Additives

Chemical recycling of PMMA waste is not the same as chemical recycling with pure virgin PMMA because it mostly contains various amounts of additives and the backbone can be of a copolymer instead of a homopolymer nature [80,81,82]. However, most investigation have dealt with homopolymers. The main contributions for more complex systems are covered in the present subsection.

For example, McNeil et al. studied the thermal degradation behavior of copolymers with MMA as a major comonomer unit and the influence of some frequently used additives on the degradation process [80,83]. The first option is that the MMA copolymer undergoes unrestricted depolymerization. This means that the degradation mechanism is unaffected by the non-MMA monomer unit, and the chains simply unzip without any hindrance. It is mentioned that this type of behavior can be observed in case the comonomer, which is also a methacrylate without ester side group decomposition during the thermal degradation. 

**Figure 17:**
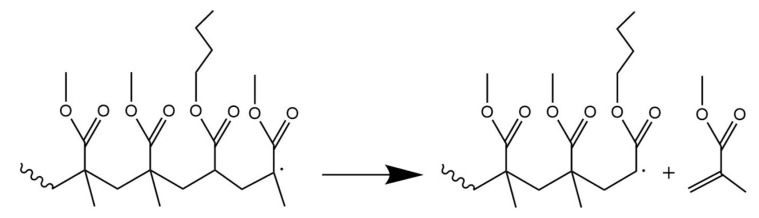
So-called blocking mechanism for unzipping (β-scission) in case n-butyl acrylate units are present in the MMA-rich backbone. The secondary radical is less stable than then typical tertiary radical so that this specific β-scission is less likely to occur.

The second option for the degradation mechanism of MMA-rich copolymers is “blocked depolymerization”. This is a mechanism in which the added comonomer upon becoming a terminal active unit is less stable so that it is likely less formed during unzipping. For example, as shown in Figure 17, acrylate units lead to secondary radicals whereas MMA to tertiary radicals. Due to this phenomenon the typical long zip length of PMMA is reduced to the MMA segment lengths in the methacrylate-acrylate copolymer. 

Alongside phenomena mentioned above, cyclisation involving an MMA unit and the adjoining comonomer unit can also take place [84,85,86]. It has been indicated that cyclization can already take place at temperatures far below the temperature needed for homolysis in the backbone or of side groups. Examples of comonomers leading to cyclisation are vinyl chloride, vinylidene chloride, methacrylic acid, acrylic acid, vinyl acetate and phenyl methacrylate. 

## 5. Lab Scale Kinetic Modeling Descriptions for Chemical Recycling of PMMA and Related Copolymers

One of the ultimate tools to improve chemical recycling is the ability to describe the product spectrum as a function of reaction time. As indicated above, many reactions can take place and, as a polymer is by definition highly stochastic as a complex product, spectrum variation is expected [61,87,88,89,90,91,92]. In this respect, kinetic modelling is an important tool as it enables to track the reaction event history of individual and lumped species. Most focus is here on lab scale conditions at which one can assume perfect mixing and temperature control. For polymer degradation this commonly implies the use of GA set-up, as covered in Figure 7a.

A large number of kinetic modelling studies have been carried out for the thermal degradation of PMMA at lab scale. Yet these have led to the publication of kinetic parameters varying widely with little to no correlation between them [93]. This is understandable as most of these parameters are apparent because they are mainly deduced from thermogravimetric experiments, which reflect overall kinetics. The lumping together of a sequence of reactions defining a degradation mechanism is implicitly performed making these models less fundamental. Hence, the field of depolymerization kinetic modelling is much less mature than the field of polymerization kinetic modelling, in which detailed elementary reaction step based models are dominant and the preferred choice [67,94,95,96,97,98,99,100,101].

In what follows, a distinction is made between chemical recycling kinetic models based on (i) conversion, (ii) (average) chain length and (iii) elementary reaction steps. To stress that certain models are extensively lumped, the term “global” is explicitly added.

### 5.1. Global Conversion Based Models 

One of the most common kinetic modelling approaches to describe thermal degradation of polymers are so-called global conversion-based models that are linked to experimental observations, as recorded with thermal analysis techniques such as TGA. A certain mass loss is here formally associated with an overall reaction (sequence), obeying an Arrhenius equation:

(1) $$k=A\;exp\left(-\frac{E}{RT}\right)$$

with *k* the “rate coefficient”, *E* the activation energy and *A* the pre-exponential factor. 

#### 5.1.1. Differential and Integrated Overall Rate Laws

The overall (differential) rate law describing a certain mass loss in the TGA experiment—thus with a given temperature (T) variation as a function of analysis time—is pragmatically written on a conversion (α) basis:

(2) $$\frac{d\alpha }{dt}=kf\left(\alpha \right)=A\;exp\left(-\frac{E}{RT}\right)f\left(\alpha \right)$$

(3) $$\frac{d\alpha }{dt}=\frac{d\alpha }{dT}.\frac{dT}{dt}$$

with *f* a to be determined function also known as the “reaction” model reflecting the overall reaction mechanism. Defining β as $\frac{dT}{dt}$ it follows that:

(4) $$\frac{d\alpha }{dT}=\frac{A}{\beta }\;exp\left(-\frac{E}{RT}\right)f\left(\alpha \right)$$

By taking the ln of Equation (4) and differentiating it over T^−1^ (at a certain α), respectively, Equations (5) and (6) can be deduced

(5) $$\ln\left(\beta \frac{d\alpha }{dT}\right)=\ln\left[A\;f\left(\alpha \right)\right]-\frac{E}{RT_{\;}}$$

(6) $${\left[\frac{d\ln\left(\frac{d\alpha }{dT}\right)}{d\left(\frac{1}{T}\right)}\right]}_{\alpha }=-{\left[\frac{E}{R}\right]}_{\alpha }+{\left[\frac{d\ln\left(f\left(\alpha \right)\right)}{d\left(\frac{1}{T}\right)}\right]}_{\alpha }$$

As shown in the second column of Table 1, several reaction models have been proposed depending on the overall mechanism operating in solid-phase degradation reaction [102]. Thermal degradation of PMMA is often found to be first-order in mass loss and thus f(α) = (1 −  α) is then formally considered [103,104].

A special case is the conversional method which assumes that the overall reaction rate at a certain extent of conversion is only a function of temperature (formally f = 1) so that Equation (6) becomes [103]:

(7) $${\left[\frac{d\ln\left(\frac{d\alpha }{dT}\right)}{d\left(\frac{1}{T}\right)}\right]}_{\alpha }=-{\left[\frac{E}{R}\right]}_{\alpha }$$

Experimentally, this simple method is often used and it is observed that E depends on α, as several reaction mechanisms can be triggered. In other words, E will (discretely) vary over the whole conversion range, depending on which (overall) reaction mechanism dominates during the degradation. 

It should be noted that the heating rates can be different so that, in practice, one has a piecewise description with different β values. Equation (5) thus needs to be updated as follows:

(8) $$\ln{\left(\frac{d\alpha }{dt}\right)}_{i}=\ln{\left(\beta \frac{d\alpha }{dT}\right)}_{i}=\ln{\left[A_{\alpha }f\left(\alpha \right)\right]}_{i}-\frac{E_{i}}{RT_{,}}$$

with the subscript *i* denoting the piecewise character. Plotting the left-hand side versus the reciprocal of the temperature allows one to determine the corresponding activation energies (*E_i_* values). For this, different non-isothermal heating experiments with different heating rates need to be performed [106].

Next to the differential method, an integral form of the overall rate equation (by convention now relating T to α instead of α to T) can be deduced as well. One obtains a constant heating rate (so constant β) starting from Equation (4):

(9) $$g\left(\alpha \right)=\int_{0}^{t}\frac{d\left(x\right)}{f\left(x\right)}$$

(10) $$g\left(\alpha \right)=\frac{A}{\beta }\;\int_{0}^{t}f\;e^{-\frac{E}{RT}}dT=\frac{A}{\beta }\;I\left(E,T\right)$$

where *I* is an auxiliary (integrated) function largely describing the conversion variation. For specific cases, a direct solution is possible, as illustrated in the last column of Table 1, but in general, numerical integration is necessary to solve the equation as there is then no analytical solution for *I*. Popular (pseudo-linearized) approximations are [103,107]:

(11) $$ln\int_{0}^{\alpha }g\left(\alpha \right)=\ln\left(\frac{k_{0}E}{k_{B}}\right)-\ln\left(\beta \;{\left(\frac{E}{k_{B}T}\right)}^{2}\right)-\frac{E}{k_{B}T}$$

(12) $$\ln\left(\frac{\beta }{T^{B}}\right)=D-C\left(\frac{E}{RT}\right)$$

in which *k*_0_ and *k_B_*, as well as *B*, *C* and *D* are parameters, with examples given in Table 2. 

Hence, by formally plotting for instance the left-hand side of Equation (12) as a function of 1/T E can be derived from the slope. Again, in practice a piecewise integration is often needed. It has been reported that the method of Kissinger Akahira and Sunose leads to very similar results as the method of Starink, whereas the method of Ozawa Flynn Wall leads to very different results. It should although be noted that many assumptions are made to end up with the equations in Table 2 and in any case overall kinetic parameters are obtained.

#### 5.1.2. Example of Thermal Degradation Process with Superposition of Overall Degradation Steps

Ferriol et al. observed a four-step degradation mechanism upon thermally degrading PMMA using TGA, considering PMMA samples with a mass average molar mass (M_m_) of 9.96 × 10^5^ and 3.50 × 10^5^ g mol^−1^ [112]. A first peak is observed at 440 K and is linked to the degradation step initiated by radical transfer to the unsaturated chain ends as described by, e.g., Manring [30] (alternative pathway in Figure 10b). The second (500 K) and the third (540 K) peak observed correspond to the degradation mechanism starting with H–H and degradation initiated by radical transfer to unsaturated ends (conventional pathway in Figure 10a), whereas the fourth peak at 625 K observed is due to a degradation initiation based on random (H–T) fission. The overall differential rate law (cf. Table 1) employed by these authors is for each step given by

(13) $$\frac{d\alpha }{dT}=\frac{A}{\beta }e^{\frac{-E_{a}}{RT}}{\left(1-\alpha \right)}^{n}$$

For the overall derivative of conversion with respect to time Equation (15) results after integration and applying Lyon’s approximation (Equation (14)), assuming that each sample contains the same relative amount of structural defects and degrades via the aforementioned degradation pathways sequentially [113]:

(14) $$-\left(\frac{e^{-\frac{E}{RT}}}{-\frac{E}{RT}}\right)+\int_{-\infty }^{-\frac{E}{RT}}\frac{e^{-\frac{E}{RT}}}{-\frac{E}{RT}}dx=\frac{e^{-\frac{E}{RT}}}{\frac{E}{RT}\left(\frac{E}{RT}+2\right)}$$

(15) $$\frac{d\alpha }{dt}=\overset{4}{\underset{i=1}{\sum }}r_{i}\left[A_{i}e^{-\frac{E_{i}}{RT}}{\left[1-\left(1-n_{i}\right).\frac{A_{i}.R}{\beta }.\frac{T^{2}}{E_{i}+2RT}.e^{-\frac{E_{a,i}}{RT}}\right]}^{\frac{n_{i}}{1-n_{i}}}\right]$$

in which *r_i_* is the relative yield regarding the mass loss of step *i* with *r_i_* < 1 and $\overset{4}{\underset{i=1}{\sum }}r_{i}$ = 1, and *E_i_*, *A_i_*, *r_i_*, and *n_i_* apparent parameters obtained by tuning the above kinetic equation to the experimentally obtained DTG curves. TGA was performed at four different heating rates (2, 5, 8 and 10 K min^−1^) and the averaged parameters are summarized in Table 3 for both sample types. It follows that the only remarkable difference between the samples with different initial *M_m_* is the reaction order. However, in case the conditions and polymer are changed a retuning is needed, highlighting the limiting potential of the overall conversion method.

For completeness it is mentioned that Korobeinichev et al. studied the thermal decomposition of high-molar mass PMMA (M_m_ = 3.5 × 10^5^ g mol^−1^) in the temperature range of 590 to 775 K [114]. The same methodology as Feriol et al. has been applied, yet the degradation of PMMA was considered to be a one-step pyrolysis reaction. An activation energy of 171.4 kJ mol^−1^ was determined and a “reaction” coefficient of 10^12.3^ s^−1^. It has also been observed that the type of inert carrier gas during the TGA experiments did not influence the kinetic parameters. 

#### 5.1.3. Single Versus Multiphase Kinetics

Fateh et al. highlighted that in kinetic models for PMMA degradation, mostly a one phase description is performed [115]. Detailed analysis of the changes in the solid phase, as well as the real time analysis of gaseous components as a function of the time, are rarely reported. Fateh et al. therefore specifically studied the gaseous phase during the thermally degradation of PMMA. Next to MMA, other components such as CO, water, methanol and CO_2_ were detected, in agreement with the discussion in Section 3.

As shown in Table 4, four (global) degradation steps (without referring to an actual detailed reaction mechanism) were formulated by these authors based on the evolution of the gaseous species observed and introducing ν as a stochiometric factor smaller than 1. First depolymerization of PMMA to form gaseous MMA (step 1) is considered. This is followed with secondary reactions and interaction with the solid matrix (step 2). This can be followed by devolatilization of various secondary products which form CO_2_ and generate carbonaceous residues (step 3), which can further undergo degradation reactions at the end of the degradative process (step 4). 

Introducing again Equation (10) for the interpretation of the TGA data individual mass-based rate laws were introduced:

(16) $${\dot{\omega }}_{i}=A.e^{-\frac{E_{a}}{RT}}.m_{i}^{n}$$

so that one can formally solve for *m_i_* values taking into account the conventions in Table 4. Here ω stands for an extension of the kinetic rate law and ν is the stoichiometric coefficient of the respective reaction step.

(17) $$\frac{dm_{PMMA}}{dt}=-\omega_{1};\;\frac{dm_{\alpha }}{dt}=\nu_{1}\omega_{1}-\omega_{2};\;\frac{dm_{\beta }}{dt}=\nu_{2}\omega_{2}-\omega_{3};\;\frac{dm_{\gamma }}{dt}=\nu_{3}\omega_{3}-\omega_{4};\;\frac{dm_{R}}{dt}=\nu_{4}\omega_{4}$$

The corresponding tuned parameters are also given in Table 4. Note that the gas contribution lowers with increasing degradation time and all steps except the last one are characterized by a similar apparent activation energy.

### 5.2. Global Chain Length Based Models 

Jellinek and Luh studied the difference between the thermal degradation of stereospecific (isotactic and syndiotactic) PMMA and atactic PMMA [116]. It has been claimed that stereospecific polymers are designed in such a manner that they do not contain unsaturated chain-ends. It has been therefore assumed that such polymers are dominantly susceptible to random H–T fission upon applying thermal degradation. These authors selected the number average chain length x_n_ as overall response for a global kinetic model, consisting of random initiation, depropagation and disproportionation, and put forward

(18) $$\ln\left(1-\frac{1}{x_{n,0}}\right)-\ln\left(1-\frac{1}{x_{n}}\right)=k_{ir}t$$

where *x_n_*_,0_ is the initial *x_n_* value and *k_ir_* is the rate coefficient for H–T fission, assuming that the average chain length is larger than the numbered averaged kinetic chain length. The latter implies that the depolymerization of a macroradical is ended by a termination event before all its monomer units are depleted via unzipping. It has been reported that the (apparent) activation energies for the iso-and syndiotactic polymers are similar and ranging between 260 and 285 kJ mol^−1^. 

### 5.3. Elementary Reaction Step Based Models

In elementary reaction step-based models, differential equations or stochastic rules are written down based on rate coefficients and actual concentrations corresponding to, ideally, a fundamental reaction scheme. These types of models are rarer and currently, in most cases, applied for other vinyl polymers than PMMA. For example, Kruse and Broadbelt used the method of moments to calculate the product spectrum in thermal degradation of polystyrene based on a single-phase kinetic model consisting of nine elementary reactions [27,60,117]. For PMMA thermal degradation, much more basic reaction schemes have been considered and often extra assumptions are made lowering the elementary nature of the actual kinetic model.

$$P_{i}\overset{k_{ir}}{\to }M_{x}^{*}+R_{i-x}\;\mathrm{random}\;\mathrm{so}\;H–T\;\mathrm{fission}$$

$$M_{x}^{*}\overset{k_{ie}}{\to }R_{x}\;\mathrm{side}\;\mathrm{group}\;\beta \;\mathrm{scission}$$

$$R_{i}\overset{k_{dp}}{\to }R_{i-1}+M\;\mathrm{depropagation}\;\mathrm{through}\;\mathrm{end-chain}\;\beta \;\mathrm{scission}$$

$$R_{i}\overset{k_{t1}}{\to }P_{i}\;\mathrm{first}\;\mathrm{order}\;\mathrm{termination}$$

$$R_{i}+R_{j}\;\overset{k_{t2}}{\to }P_{i}+P_{j}\;\mathrm{termination}\;\mathrm{by}\;\mathrm{disproportionation}$$

Figure 17: Simplified reaction scheme for elementary reaction based kinetic modeling of thermal degradation of PMMA [27], with *k_ir_*, *k_ie_*, *k_dp_*, *k_t1_* and *k_t2_* the rate coefficients for random initiation, side group β scission, depropagation through end-chain β scission, and first and second order termination reactions. First order termination must be seen as a formal reaction. For side group β scission, the extra radical is assumed for simplicity as unreactive (not shown).

For example, Kashiwagi et al. proposed a kinetic model in which they made the distinction between primary and tertiary radicals [27]. The model describes the degradation of PMMA with one initiation type, one depropagation type and two types of termination, as depicted in Figure 17. The initiation reaction stands for the degradation of a PMMA polymer molecule with chain length i via head-tail-fission with the formation of a primary radical (M*), with chain length x and a tertiary radical (R) with chain length i–x. The primary radical formed is assumed to undergo side-group β-scission to form a tertiary radical which can consequently depropagate with the formation of MMA (M). It has to be noted that the extra formed radical of the side group β scission is assumed unreactive, which is a strong simplification. Termination is assumed to occur via two different mechanisms. One is a (formal) first order termination in which the macroradical is transformed into a neutral (polymer) molecule. The second termination reaction is the disproportionation between two macroradicals. The deterministic model gave a good fit with the obtained experimental results. The overall activation energy was also determined by Kissinger’s method (Table 2) [118] and found consistent with the kinetic model. 

Furthermore, Holland et al. studied the kinetics and mechanism of thermal degradation of PMMA [93,119]. To further simplify the kinetic description these authors applied the steady-state approximation for the calculation of the radical concentration $\left[R^{.}\right]$, starting from a basic reaction scheme [120]:

(19) $$\frac{d\left[R^{.}\right]}{dt}=\left(\frac{k_{i}}{DP}+2k_{i}^{’}\right)\frac{\rho }{MM}-r_{t}=0$$

In Equation (19), DP stands for the degree of polymerization (thus xn), *ρ* stands for the polymer density, *k_i_* for the first order rate coefficient for initiation by head-tail fission, *k’_i_* for the first-order rate coefficient for initiation by chain-end fission and *r_t_* for the termination rate. *MM* stands for the molar mass of a monomer unit. In a first instance, a formal distinction was made between first and second order termination, leading to two rates of radical concentration variation:

(20) $$r_{t,1}=k_{t}^{,}\left[R^{.}\right]$$

(21) $$r_{t,2}=2k_{t}{\left[R^{.}\right]}^{2}$$

Note that formally a factor two is added for the latter equation or implicitly the same chain length is assumed for the participating radicals. For a formal first-order termination this implies following observed rate coefficient:

(22) $$k_{obs}=\left(\frac{k_{i}}{DP}+2k_{i}^{,}\right)\frac{k_{dp}}{k_{t}^{,}}$$

in which *k_dp_* is the first-order rate coefficient for depropagation. A (linear) plotting of *k_obs_* as a function of 1/DP for a range of mass losses allows thus to assess specific rate coefficients. For bimolecular termination it similarly holds that:

(23) $$k_{obs}=\left(\frac{k_{i}}{DP}+2k_{i}^{,}\right)\frac{k_{dp}MM^{1/2}}{{\left(2k_{t}\rho \right)}^{1/2}}$$

Degradation experiments were analyzed using thermal analysis-Fourier transform infrared spectroscopy (TA-FTIR). Good linearity between *k_obs_* and 1/DP was observed by Holland et al. up to 20% conversion. At 693 K, more char formation was, however, observed than at 634 K, which indicates that random side group fission, as not considered in the kinetic model, is more thermally activated than the other initiation reactions. The slopes of both isotherms are similar, yet the intercept at 693 K increased by a factor of two compared to the intercept at 634 K, further indicating that with increasing temperature, the involvement of side-group fission increases. It was further observed that degradation was initiated both by chain end fission and random chain fission. This differs from the observations made by previous studies for which at low degradation temperatures, chain end fission was the only dominant mechanism. The corresponding activation energies are respectively 150 ± 25 and 210 ± 40 kJ mol^−1^. This shows that it easier to break a specific end-group (defect) than a typical bond in the polymer backbone. 

At higher temperatures, it was put forward that an additional process is taking place which had a greater effect on samples with a higher initial DP. This process, with rate coefficient $k_{v}$, is depropagation ending by volatilization of a small chain end radical, thus highlighting the multiphase character, as also introduced above. This makes this third rate of termination dependent upon the number of chain ends thus 1/DP :

(24) $$r_{t,3}=k_{v}\left[R^{.}\right]\left(\frac{1}{DP}\right)$$

This implies for instance that Equation (22) becomes:

(25) $$k_{obs}=\left(k_{i}+2k_{i}DP\right)\frac{k_{dp}}{k_{v}}$$

Furthermore, Da Ros et al. studied the effect of PMMA crosslinks on the depolymerization kinetics [121], selecting ethylene glycol dimethacrylate (EGDMA) as crosslinker. For this, a kinetic model was developed, containing a limited number of overall reactions able to predict non-isothermal TGA experiments. The model was also validated against isothermal TGA experiments at 673 K. A distinction has been made between two types of depolymerization reactions, one being a set of independent reactions and one being a set of consecutive reactions, as can be seen in Table 5 (left vs. right column). It was concluded that the consecutive reaction model gave better results than the model with independent reactions. For the two consecutive degradation steps, the activation energies and pre-exponential factors were determined, with activation energies of 186 and 56 kJ.mol^−1^ and pre-exponential factors of 2.0 × 10^13^ and 4.4 × 10^2^ min^−1^. Note that this model is a mix of an overall and elementary reaction step driven model, but preference was given to list it in the section of elementary driven models as it could be extended in that way in future work.

Smolders and Baeyens studied in addition the thermal degradation of PMMA in a fluidized bed [122], considering the reaction mechanisms and associated rate laws postulated by Barlow et al. and as shown in Table 6 [123]. Here RM stands for the volumetric production reaction rate of monomer formally assuming a single-phase reaction mixture. The activation energies of the overall depolymerization reaction mechanisms, as described in Table 6, were assessed from the activation energy of the elementary reaction steps as shown in Table 7. 

Finally, Staggs [124,125] developed a mathematical model to analyze the evolution of a population of molecules undergoing end-chain fission and recombination. Furthermore, volatilization of certain species was incorporated so that comparison with experimental thermogravimetric studies could be made. The model was capable of predicting the thermogravimetric experiments involving commercial PMMA sheets. The results were also compared with those obtained via Monte Carlo simulations. Unfortunately, no further information was given on how the Monte Carlo model was designed. 

## 6. Reactor Technologies

A next logical step from lab scale kinetic analysis is the identification of the best reactor technology. As explained below, several reactor technologies have been developed for PMMA chemical recycling. Attention is paid to patented reactor technologies, with the discussion complemented with supportive research results as published in the open literature.

### 6.1. Molten Metal-Bath Reactor 

**Figure 18:**
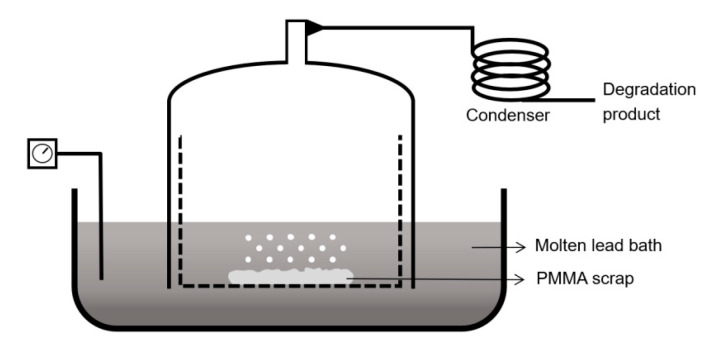
Molten-lead bath technology patented by Segui et al. [126].

To date, the most applied technology in industry is the molten-lead bath process [126]. This process dates back to 1958 when it was patented by Segui et al. [126]. The patent of which, the main principle depicted in Figure 18, describes the use of a bath filled with molten lead operated at a temperature between 725 and 775 K. The PMMA scrap is fed to the reactor vessel and comes in contact with the lead once immersed in the molten lead bath. The gases formed are collected and condensed. The obtained monomer (MMA) has a claimed purity of around 94 m%, yet it is reported that even a purity of up to 98 m% can be reached [127]. Other metals and salts have been investigated [128,129], but because of the low volatility of lead at the operating temperature and the high yield of MMA, lead melt is the most preferred heat transfer medium. One of the main disadvantages is that carbonaceous side products are formed in small amounts, which accumulate at the surface. The formation of this lead containing side product also depends on the purity of the PMMA scrap used. In other words, the molten-lead bath process is ecologically more relevant upon working with pure PMMA scrap.

### 6.2. Counter Current Reactor 

**Figure 19:**
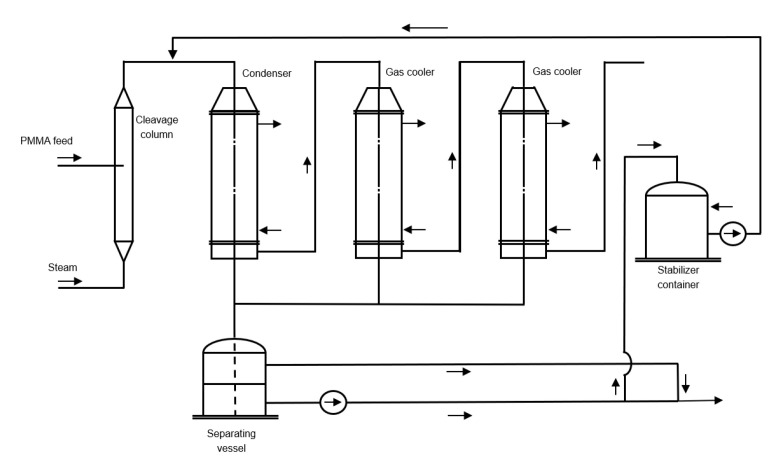
Configuration for the chemical recycling of PMMA as developed by Mannsfeld et al. based on counter current reactor technology [130].

Mannsfeld et al. patented a technology in which they made use of a counter current reactor design in which it is favored that the gaseous heat transfer agent is in counter current with the fed finely grounded polymer [130]. Granulated PMMA scrap, with a preferred average diameter of maximum 1.5 mm is fed to a column at a certain height, while steam is fed at the bottom. Because of the smaller particle size, a better heat transfer efficiency between the effluent and the PMMA particles is obtained. It is mentioned that a temperature of 825 to 1065 K is desired for the cleavage of the larger PMMA particles, which takes place at the lower region of the cleavage column. The cleavage of the smaller particles takes places in the upper region of the cleavage column at a temperature of 675 to 825 K. The lower temperature at the top of the cleavage column is due to the wind sifting action occurring therein. By making use of steam, it is relatively easy to remove the solvent (water) from the MMA product in the consecutive purification section. The purification section exists out of a series of condensers and a separating vessel, as shown in Figure 19. There is no evidence that such a process has ever been built. 

### 6.3. Paddle Reactor

**Figure 20:**
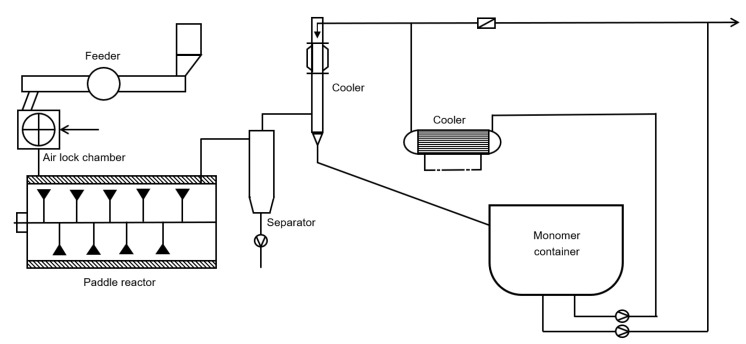
Patented depolymerization technology by Schola et al. in which use is made of a paddle reactor [131].

Schola et al. published a patent in which a technology is described for the depolymerization of PMMA by making use of a paddle reactor, as shown in Figure 20 [131]. The polymer feed is first brought under an inert atmosphere before added to the reaction chamber. The paddle reactor is also operated under an inert atmosphere at a temperature ranging between 525 to 875 K. Inert, chromium- and nickel containing steel spheres are used to enhance the degradation process as a heat transfer medium. The formed MMA containing gas is entrained from the reactor via a monomer gas line to a separator (cyclone). The MMA containing gas is then send to a cooler unit, in which it is cooled by bringing it in contact with cooled and condensed MMA. The cold MMA is fed at the top of the cooling column. Condensed MMA is partly recirculated for cooling. 

### 6.4. Fluidized Bed Reactor

The majority of academic reactor studies focused on the use of a fluidized bed reactor setup, as it is generally accepted that the good thermal energy transfer in a fluidized bed aids to a better selectivity towards MMA conversion. It has to be mentioned that in the literature, to some extent, attempts have been made to describe a fluidized bed reactor by a continuous stirred tank reactor. A design procedure has, for instance, been outlined and illustrated by Smolders and Baeyens [122]. The high interest of the fluidized bed reactor technology also follows from the several patents that have been proposed, as also explained below.

**Figure 21:**
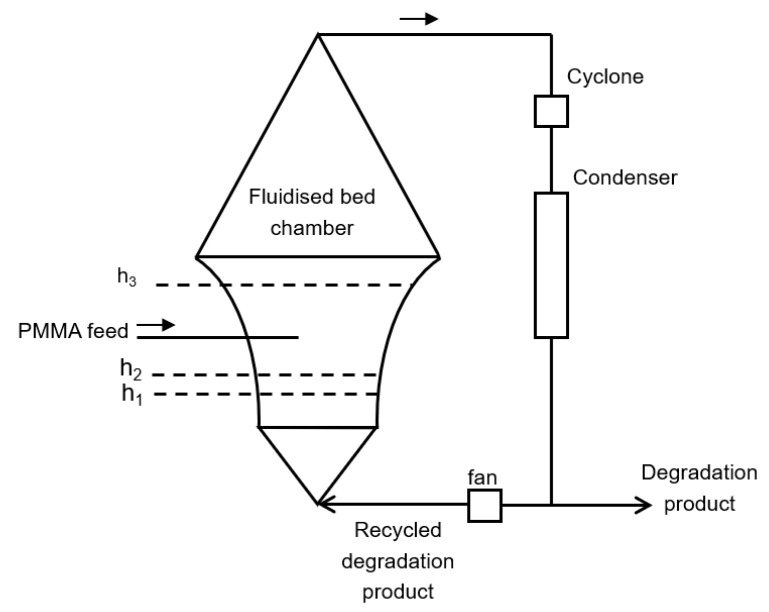
Fluidized bed reactor technology as proposed by Vaughan et al. [127].

For example, Vaughan et al. describe a fluidized bed degradation reactor which is operated at relatively low temperature [127]. It is operated below the self-ignition temperature of the monomer, which is 700 K. The technology allows particles to remain within the body of the bed, where the necessary high heat transfer can be maintained, without foaming. The fluidized bed is preferred to contain an inert material such as silicon carbide, silica or alumina. Special about the fluidized bed is the design of the fluidized bed chamber, the cross-section of which increases upwardly, as can be seen in Figure 21, and that the fluidization is driven by recycling gaseous products. Here, h1 resembles the height of the bed when static, h2 when fluidized by an inert gas and h3 when fluidization is additionally driven by gaseous products. 

**Figure 22:**
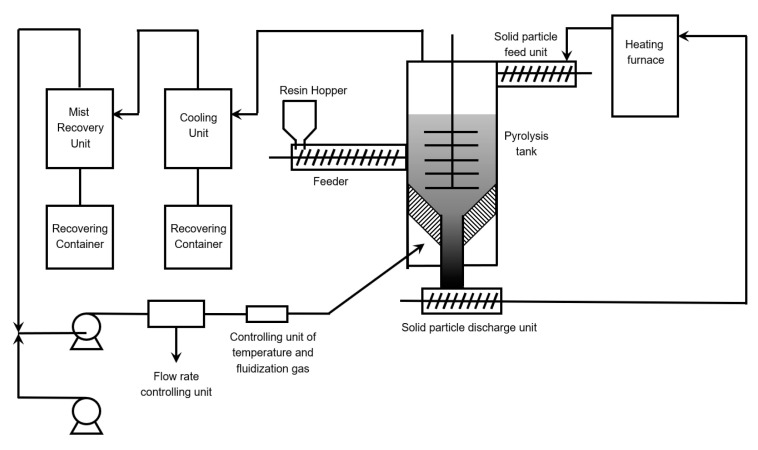
Alternative fluidized bed reactor technology as patented by Sasaki et al. [132].

Sasaki et al. patented a technology also makes use of a fluidized bed reactor for the thermal degradation of PMMA [132]. As shown in Figure 22, sand was used as the heat transfer medium and was fed at the top of the column, entering at around 873 K. Recirculation of the sand happened by collecting it at the bottom of the fluidized bed reactor. Before the sand is re-fed, the entrained resin remainders are burned off and the sand (heat carrier) is reheated. Polymer resin was fed at a certain height and on entering the reactor homogeneously mixed via mechanical agitation. The formed gaseous products are together with the fluidization gas removed from the reactor. Via condensation and a demister, the depolymerization product spectrum is collected and the fluidization gas recirculated. These authors claimed that the yield of the recovered liquids was around 95%, with an MMA purity of 96%. 

Furthermore, Kaminsky et al. reported the use of a laboratory plant with a capacity of 3000 g h^−1^ [133,134]. Small milled PMMA particles were fed to the fluidized bed reactor and degraded at temperatures ranging between 875 and 1175 K. It has been observed that beyond 825 K, the formation of gaseous product increased significantly, reaching 42 m% at 865 K. The main component in the liquid pyrolysis oil was MMA with 98.6 m% purity at 725 K and 98.3 m% at 765 K. Furthermore, contaminated PMMA has been tested considering filled and colored waste PMMA, which also yielded a purity of 98.6 m%. Only small amounts of carbon formation were observed during the experiments, which were the highest for the real waste samples with an amount of 0.51 m%. In general, it was concluded that the PMMA conversion declined with increasing temperature. It has been also observed that the overall activation energy of the depolymerization was around 102 kJ mol^−1^ at 675 K. 

More recently Kaminsky et al. studied the monomer recovery from filled PMMA [135]. Silica filled PMMA has been pyrolyzed and yielded 90 m% of the MMA monomer. Upon pyrolyzing aluminum trihydroxide (ATH) filled PMMA, which is added as flame retardant and as white pigment, water is formed during its thermal degradation. Only 58 m% MMA monomer has been obtained compared to the 97 m% upon pyrolyzing virgin PMMA. Hydrolysis products such as methacrylic acid, methanol and isobutyric acid have been reported to be the other main components. It has been indicated that the high amount of aluminum components does not possess a catalytic influence on the hydrolysis reactions taking place during the thermal degradation. Furthermore, Kang et al. observed that the main thermal degradation of PMMA takes place between 625 and 675 K with fluidized bed reactor technology [136]. PMMA containing MA and ethyl acrylate (EA) units have been also pyrolyzed and yielded 98 m% MMA. Furthermore, automobile tail light lenses and light guiding plates have been pyrolyzed with claimed yields of 93 m% of MMA. 

**Figure 23:**
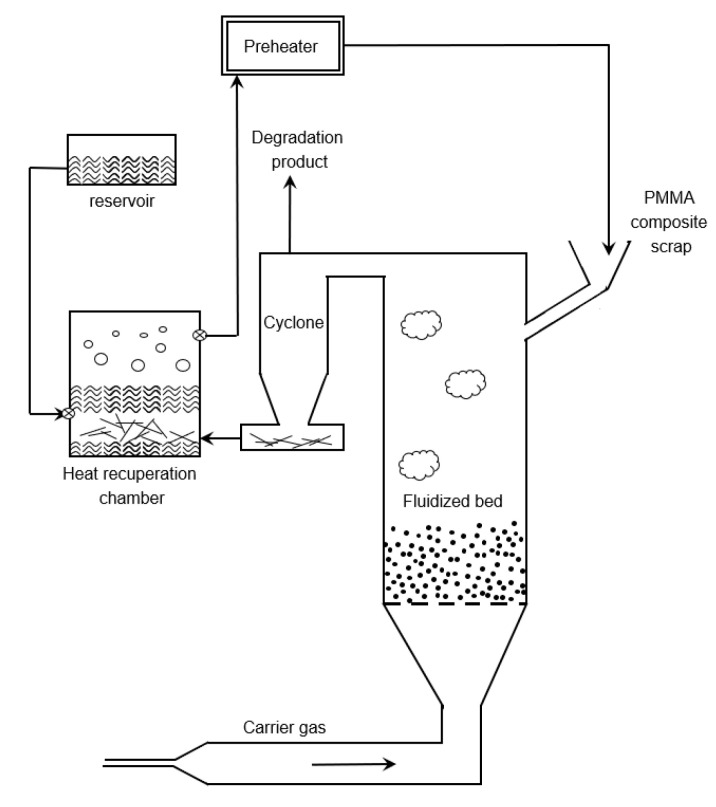
Degradation process described by Dubois et al. in which us is made of a fluidized bed for the degradation of PMMA-fiber-based composites [137].

Dubois et al. described a fluidized bed reactor in which PMMA-fiber composites are fed via a hopper [137], as can be seen in Figure 23. A particle size of 25 mm is desired for the fluidized bed. No specific fluidization medium has been mentioned as sand, ceramic particles, metallic particles and other materials can be used. Fluidization is realized by introducing an inert warm gas. Thermal degradation is performed between 573 and 623 K. The gaseous product from the fluidized bed reactor is entrained by the inert fluidization gas and sent through a cyclone which separates the solids from the gas. It is claimed that the solid particles from the polymer composite, which are assumed not to degrade, are also entrained by the gaseous flow leaving the reactor. The remaining gaseous product is obtained at the head of the cyclone. Before the remaining solid particles of the composite are retrieved from the process, the thermal energy is recovered. This is realized by making use of a thermal liquid which transfers the heat from the remaining solid composite fraction to the feed of the reactor, preheating the feed. It is noted that the efficiency of the energy recuperated varied with the fiber content of the materials.

Finally, Lopez et al. [7] studied the pyrolysis of PMMA in a conical spouted bed reactor as an alternative to the widely reported conventional fluidized bed reactor, taking into account advantages such as the smaller pressure drop over the bed and the simpler design [138]. A copolymer of MMA and EA (<10 m%) has been used as feed for the pyrolysis experiments. The main product in the pyrolysis oil collected contained mainly MMA. Again, the negative effect of the temperature increase has been observed as more gaseous products are formed with increasing temperature. Furthermore, an overall activation energy off around 165 kJ mol^−1^ has been observed.

### 6.5. Extrusion Based Reactors

Several patents in which use is made of extrusion technology have been assigned [139,140,141,142]. The extruder is used to heat up and initiate the thermal degradation of PMMA. Heat is additionally generated because of mechanically induced shearing [139,143]. The advantage of an extruder instead of a fluidized bed reactor setup is the easier operating of the overall process. For example, graphitization is avoided, as one of the disadvantages of a fluidized bed reactor with quartz sand as fluidized material is that the fluidized material can become graphitized. The soot can come off the grains as well and be entrained with the gas stream so that extra filtration systems are required, which are not needed for extrusion technology. Often, the MMA is withdrawn via a degassing bell and condensed later on. The MMA contents of the collected condensate are reported to be between 89 and 97 m%. 

**Figure 24:**
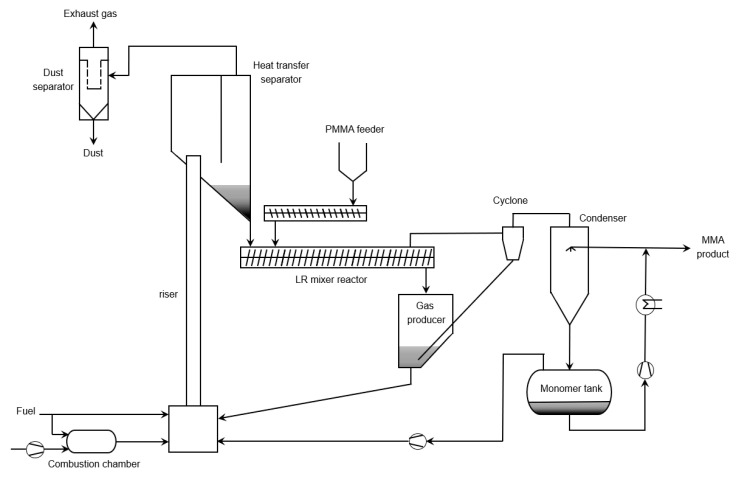
Extrusion technology for depolymerization of PMMA as patented by Weiss et al. [139].

Weiss et al. patented extrusion technology in which they claimed the degradation process could be easily operated at industrial scale [139]. They put forward that the technology is able to perform residue free degradation of PMMA. Free of residues implies here that one avoids the formation of deposits in the reactor so that continuous operation is ensured. It is known that the heating of the PMMA is affected by the shell wall quality inside the extruder. However, with increasing plant size, the ratio of wall surfaces area to reactor volume declines, so that, for larger plants, the extruder has to be set at a much higher temperature in order to decompose sufficiently amounts of PMMA. However, this heating can lead to local hotspots causing the amount of side products formed to increase, as highlighted in Section 3. Because of this issue when sizing up to an industrial plant, Weiss et al. (Figure 24) made use of a heat transfer medium. In the reactor, polymeric material is brought into contact with a hot mechanically fluidized solid. Next to the heat transfer purpose, the fine-grained heat-transfer medium fulfils a cleaning purpose by scrubbing away the residues inside the reactor so that the by-products are continuously discharged and agglomeration of by-products inside the reactor is prevented. The by products are subsequently removed from the heat-transfer medium by burning them off in an oxygen-rich environment. The degradation is preferably performed between 575 and 725 K and the regeneration of the heat-transfer medium is done between 775 and 1025 K. The collected depolymerization products in the gas phase are directly cooled by recirculating cooled product and the condensed products are then sent to a separating vessel with the non-condensable gasses brought to the heat-transfer medium regeneration section. 

**Figure 25:**
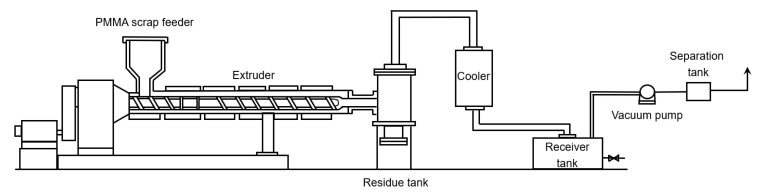
Extrusion technology as patented by Japan Steel Works [140].

As shown in Figure 25, Japan Steel Works developed extrusion reactor technology as well, but without the use of an additional heat-transfer medium [140]. Both single- and twin-screw designs can be applied. The PMMA feed is placed in the hopper and by the rotating screw pushed through the extruder. Temperatures inside the extruder are reported to be between 675 and 875 K. The novelty of the design lies in extrusion of the formed vapors toward the end of the extruder, as opposed to a direct extraction via multiple vents. As the molten polymer acts as a sort of plug, backflow of the vapors is averted. The solid and gaseous products are pushed inside a residue tank where the vapor product is separated from the solid residue formed during the thermal degradation of PMMA. The gaseous products are then condensed in a consecutive cooler. 

The molten metal-bath technology is exploited in Europe and several other countries. The process produces a good quality MMA, from high quality PMMA waste, but is inappropriate to recycle the lower quality end-of-life PMMA due to the production of higher amount of solid residues contaminated with the metal. Hence, the most promising reactor technologies are the fluidized bed reactor and extrusion technology. The technology selected in the EU project MMAtwo is based on a twin-screw extruder, which operates in a continuous mode. That technology is able to handle materials which are going to produce high amount of solid residue. The low residence time in the depolymerization reactor avoids the production of secondary degradation products. However, a better linkage with the chemistry scale is required preferably via dedicated elementary reaction step driven kinetic models.

## 7. Conclusions

In contrast to most other vinyl polymers, pure PMMA can be almost quantitatively depolymerized to monomer, provided that the correct thermal degradation conditions are identified and the initial chemical backbone structure is known. For waste PMMA the latter is non-trivial, explaining why experimental protocols for the thermochemical recycling of pure PMMA cannot be directly translated to the circularity concept. Today, still a limited understanding exists regarding the decomposition chemistry of PMMA and its related copolymers, but some continuous processes are already operational. A key challenge is the realization of adequate heat transfer to facilitate chemical transitions toward the fast and high yield formation of MMA. In an industrial framework, fluidized bed and extrusion-based reactor technologies seem the most promising. 

Even though the thermal degradation of PMMA has been reported many decades ago much uncertainty remains about the exact reaction mechanism(s) for a given PMMA type and set of reaction conditions. This can be explained by the main focus on overall or global degradation kinetics, as covered by experimental techniques such as DTG/TGA. Preference should be given to a kinetic understanding at the elementary reaction level, as chemical degradation reactions are related to the presence of specific structural defects and functional groups in the originally synthesized PMMA. In this respect, the development of fundamental kinetic models is indispensable, as they offer more quantitative information in view of advanced process design and do not rely on heuristics, empiricism and polynomial fitting, making them flexible for a variation in the starting polymer composition. 

The number of kinetic models based on elementary steps remains, however, limited, despite being an insurmountable step to design reactors for chemical recycling of PMMA homo- and copolymers. Mass transport phenomena, such as diffusional limitations and evaporation, are also often neglected, yet disguise the chemical kinetics of the degradation process, leading to almost exclusively apparent activation energies being reported in the scientific literature. Nearly all models are homogeneous in nature, discarding the co-existence of a solid, a melt and a gas phase, and the transitions between them. Moreover, the presence of additives in PMMA can further affect reactivity and selectivity.

Hence, future research should be directed toward the development of multi-scale modeling tools for chemical recycling of PMMA, covering a detailed reaction scheme, interphase mass transport and an appropriate reactor model, all of this in combination with experimental validation according to detailed analysis of time dependent degradation product spectra. 

## Figures and Tables

**Table 1:** Overview of typical reaction models for global conversion based models in solid-phase degradation; f: differential; g: integrated cf. Equation (9) [102,103,105].

Mechanism	f(α)	g(α)
Power law (P2)	2 α^1/2^	α ½
Power law (P3)	3 α^2/3^	α ^1/3^
Power law (P4)	4 α ^3/4^	α ¼
Avarami-Erofe’ve (A2)	2(1 − α)[−ln(1 − α)] ^1/2^	[−ln(1 − α)]^1/2^
Avarami-Erofe’ve (A3)	3(1 − α)[−ln(1 − α)] ^2/3^	[−ln(1 − α)]^1/3^
Avarami-Erofe’ve (A4)	4(1 − α)[−ln(1 − α)] ^3/4^	[−ln(1 − α)]^1/4^
Contracting sphere (R2)	2(1 − α) ^1/2^	[1 − (1 − α)^1/2^]
Contracting sphere (R3)	3(1 − α) ^2/3^	[1 − (1 − α)^1/3^]
One-dimensional diffusion	1/2 α	α^2^
two-dimensional diffusion	[−ln(1 − α)] ^−1^	[(1 − α)ln(1 − α)] + α
three-dimensional diffusion	3(1 − α)^2/3^/[2(1 − (1 − α)^1/3^)]	[1 − (1 − α)^2/3^]^2^
Ginstling-Brounshtein	3/2((1 − α)^−1/3^ − 1)	1 − (2α/3) − (1 − α)^2/3^
First-order	1 − α	−ln(1 − α)
Second-order	(1 − α)^2^	(1 − α)^−1^ − 1
Third-order	(1 − α)^3^	[(1 − α)^−2^ − 1]/2

**Table 2:** Approximations for integral from, starting from Equations (11) and (12) (no piecewise integration here for simplicity).

Method	B	C	Expression	Reference
Ozawa Flynn Wall	0	1.052	$\ln\left(\beta \right)=D-1.052\left(\frac{E}{RT}\right)$	[108,109]
Kissinger Akahira Sunose	2	1	$\ln\left(\frac{\beta }{T²}\right)=D-\frac{E}{RT}$	[110]
Starink	1.92	1.0008	$\ln\left(\frac{\beta }{T^{1.92}}\right)=D-1.0008\left(\frac{E}{RT}\right)$	[111]

**Table 3:** Apparent kinetic parameters for the thermal degradation of PMMA with a low (3.50 × 10^5^ g mol^−1^) and high (9.96 × 10^5^ g mol^−1^) mass average molar mass (M_m_) by Ferriol et al.; tuning based on Equation (12) with several heating rates.

Step	M_m_	E_i_ (kJ mol^−1^)	Log(A_i_)	n_i_	r_i_
1	Low	182.5 ± 3.4	20.817 ± 0.376	2.06 ± 0.46	0.041 ± 0.012
	High	190.0 ± 1.2	21.887 ± 0.211	1.90 ± 0.31	0.030 ± 0.011
2	Low	265.7 ± 10.8	27.828 ± 0.519	7.59 ± 1.82	0.039 ± 0.027
	High	263.7 ± 5.2	27.001 ± 0.720	2.19 ± 0.31	0.019 ± 0.015
3	Low	124.8 ± 3.3	10.560 ± 0.102	1.53 ± 0.21	0.143 ± 0.032
	High	118.9 ± 1.6	10.657 ± 0.085	1.30 ± 0.19	0.337 ± 0.033
4	Low	200.4 ± 0.3	16.059 ± 0.023	1.21 ± 0.04	0.777 ± 0.041
	High	199.2 ± 04	15.976 ± 0.023	1.21 ± 0.06	0.614 ± 0.017

**Table 4:** Overall steps and apparent kinetic parameters proposed by Fateh et al. considering the multiphase nature of the chemical recycling; fitting based on Equations (13) and (14); R: residue [115]. MMA is formally part of the gas phase contributions.

Step		Log(A_i_)	E_i_	n_i_	$\nu_{i}$
1	$PMMA\;\to \nu_{1}\;PMMA_{\alpha }+{\left(1-\nu_{1}\right)}_{gas}$	16.5	158	3.9	0.98
2	$PMMA_{\alpha }\;\to \nu_{2\;}PMMA_{\beta }+{\left(1-\nu_{2}\right)}_{gas\;}$	10.8	154	0.85	0.60
3	$PMMA_{\beta }\;\to \nu_{3}\;PMMA_{\gamma }+{\left(1-\nu_{3}\right)}_{gas}$	17.0	161	1	0.17
4	$PMMA_{\gamma }\;\to \nu_{4}\;R+{\left(1-\nu_{4}\right)}_{gas}$	14.3	215	0.83	0.02

**Table 5:** Reactions considered for chemical degradation of crosslinked PMMA, as done by Da Ros et al. [121].

Model with Independent Reactions	Model with Consecutive Reactions
$P_{1}\overset{k_{1}}{\to }V$	$P_{1}\overset{k_{4}}{\to }P_{2}+V$
$P_{2}\overset{k_{2}}{\to }V$	$P_{2}\overset{k_{5}}{\to }P_{3}+V$
$P_{3}\overset{k_{3}}{\to }V$	$P_{3}\overset{k_{6}}{\to }V$

**Table 6:** Simplified rate laws reported by Barlow et al. [123] ^a^.

Mechanism	Reaction Steps	Reaction Rates	Initial Conditions
1	Random fission followed by complete depropagation	$R_{M}=2k_{ir}x_{0}^{2}c_{p}$	T = 700–730 K DP = 105–570
2	Random fission, depropagation and termination by disproportionation	$R_{M}=2k_{dp}\sqrt{\frac{k_{ir}x}{k_{td}}}c_{p}^{1/2}$	T = 700–730 K DP = 570–2970
3	End-chain fission, depropagation and termination by disproportionation	$R_{M}=k_{dp}\sqrt{\frac{2k_{ie}}{k_{td}}}c_{p0}^{1/2}$	T = 600–670 K

^a^*k_ir_* stands for the rate coefficient for random fission, *k_ie_* for the rate coefficient for end-chain fission, *k_dp_* for the rate coefficient for depropagation and *k_td_* for the rate coefficient for termination by disproportionation. *c_p_* is the polymer concentration; x stands for the number average chain length and *x_0_* the initial number average chain length prior to degradation.

**Table 7:** Theoretical determination of overall activation energies by Smolders and Baeyens [122], based on the reaction schemes and rate laws in Table 6.

Mechanism	Overall Rate Coefficient	Activation Energy ^a^
1	$k_{ir}$	$E_{ir}=287\;\mathrm{kJ}.{\mathrm{mol}}^{-1}$
2	$k_{dp}\sqrt{\frac{k_{ir}}{k_{td}}}$	$E_{dp}+0.5\left(E_{ir}-E_{td}\right)=190{\;\mathrm{kJ}\;\mathrm{mol}}^{-1}$
3	$k_{dp}\sqrt{\frac{k_{ie}}{k_{td}}}$	$E_{d}+0.5\left(E_{ie}-E_{td}\right)=75{\;\mathrm{kJ}\;\mathrm{mol}}^{-1}$

^a^ with E_td_ = 12 kJ mol^−1^, E_dp_ = E_ie_ = 54 kJ mol^−1^ and a dissociation energy of 275 kJ mol^−1^.
